# Exploring with [^18^F]UCB-H the *in vivo* Variations in SV2A Expression through the Kainic Acid Rat Model of Temporal Lobe Epilepsy

**DOI:** 10.1007/s11307-020-01488-7

**Published:** 2020-03-23

**Authors:** Maria Elisa Serrano, Mohamed Ali Bahri, Guillaume Becker, Alain Seret, Charlotte Germonpré, Christian Lemaire, Fabrice Giacomelli, Frédéric Mievis, André Luxen, Eric Salmon, Bernard Rogister, Robrecht Raedt, Alain Plenevaux

**Affiliations:** 1 GIGA, CRC in vivo imaging, University of Liège, Allée du 6 Août, Building B30, Sart Tilman, 4000 Liège, Belgium; 2 Department of Neuroimaging, Institute of Psychiatry, Psychology and Neuroscience, King’s College London, London, SE5 9NU UK; 3 Radiobiology Unit, SCK•CEN, Belgian Nuclear Research Centre, 2400 Mol, Belgium; 4 4Brain lab, University of Ghent, 9000 Ghent, Belgium; 5 Nucleis, Allée du 6 Août, Building B30, Sart Tilman, 4000 Liège, Belgium; 6 Neurology Department, CHU, Academic Hospital, University of Liège, 4000 Liège, Belgium; 7 GIGA-Neurosciences, University of Liège, Avenue Hippocrate, 15, 4000 Liège, Belgium

**Keywords:** Temporal lobe epilepsy, Kainic acid, SV2A, [^18^F]UCB-H, EEG, SV2A immunofluorescence, T2 MRI

## Abstract

**Purpose:**

The main purpose of this study was to understand how the positron emission tomography (PET) measure of the synaptic vesicle 2A (SV2A) protein varies *in vivo* during the development of temporal lobe epilepsy (TLE) in the kainic acid rat model.

**Procedures:**

Twenty Sprague Dawley male rats were administered with multiple systemic doses of saline (control group, *n* = 5) or kainic acid (5 mg/kg/injection, epileptic group, *n* = 15). Both groups were scanned at the four phases of TLE (early, latent, transition, and chronic phase) with the [^18^F]UCB-H PET radiotracer and T2-structural magnetic resonance imaging. At the end of the scans (3 months post-*status epilepticus*), rats were monitored for 7 days with electroencephalography for the detection of spontaneous electrographic seizures. Finally, the immunofluorescence staining for SV2A expression was performed.

**Results:**

Control rats presented a significant increase in [^18^F]UCB-H binding at the last two scans, compared with the first ones (*p* < 0.001). This increase existed but was lower in epileptic animals, producing significant group differences in all the phases of the disease (*p* < 0.028). Furthermore, the quantification of the SV2A expression *in vivo* with the [^18^F]UCB-H radiotracer or *ex vivo* with immunofluorescence led to equivalent results, with a positive correlation between both.

**Conclusions:**

Even if further studies in humans are required, the ability to detect a progressive decrease in SV2A expression during the development of temporal lobe epilepsy supports the use of [^18^F]UCB-H as a useful tool to differentiate, *in vivo*, between healthy and epileptic animals along with the development of the epileptic disease.

**Electronic supplementary material:**

The online version of this article (10.1007/s11307-020-01488-7) contains supplementary material, which is available to authorized users.

## Introduction

Worldwide, around 50 million people suffer from epilepsy, a neurological chronic disorder characterized by recurrent and unprovoked seizures [1]. Nevertheless, approximately 30 % of the patients are unresponsive to the available treatments. Thus, studying the underlying pathophysiology of this disease is essential for the discovery of more efficient treatments [2, 3]. In this regard, the development and preclinical evaluation of new positron emission tomography (PET) radiotracers has provided new longitudinal insights on the neurobiological processes affected by epilepsy [4, 5]. Among them, two have been extensively investigated: (1) alterations in the glucose metabolism of the brain, quantified through [^18^F]-fluoro-2-deoxy-D-glucose ([^18^F]FDG) radiotracer [6, 7], and (2) neuroinflammatory processes, detected with different translocator protein (TSPO) markers [8–10]. Over the last few years, a third potential biomarker has drawn attention to the scientific community: (3) the determination of synaptic density through the evaluation of the synaptic vesicle 2A (SV2A) protein [11–14].

SV2A is a transmembrane glycoprotein involved in the synaptic transmission, whose expression in the brain is ubiquitous [15–17]. Genetically, the homozygous mutation of this protein has been associated with intractable epilepsy, growth retardation, and premature death, both in animals [18, 19] and in humans [20]. Furthermore, different studies have identified the SV2A protein as the target of one of the most effective antiepileptic drugs: levetiracetam [17, 21]. Therefore, an important link between the altered SV2A expression and/or function and the epileptic process is expected. This potential link has encouraged the continuous development of different radiotracers to perform the *in vivo* evaluation of this protein. Among them, two have been widely characterized: [^18^F]UCB-H [22–26] and [^11^C]UCB-J [14, 27, 28].

In this paper, we performed the first *in vivo* longitudinal study of variations in SV2A expression in the kainic acid (KA) rat model of temporal lobe epilepsy (TLE). This model reproduces the different phases of the epileptogenic process in patients [29, 30]: early phase (initial precipitating insult or *status epilepticus* (*SE*)), latent phase (seizure-free period), transition phase (onset of first spontaneous seizures), and chronic phase (recurrent and spontaneous seizures). Variations in SV2A levels, measured *in vivo* with [^18^F]UCB-H radiotracer, were quantified in these four phases. Additionally, at the end of the PET scans, we tested for a possible correlation between variations in SV2A expression and the occurrence of electrographic seizures. Finally, we performed SV2A immunofluorescence on brain slices of the same animals, to confirm the [^18^F]UCB-H *in vivo* results.

## Materials and Methods

### Animals

Twenty Sprague Dawley CD male rats, from 4 to 5 weeks old, were obtained from Janvier Laboratories (France). The minimum sample size for the experiment (*n* = 4) was estimated with the G*Power software (G*Power, v 3.0), setting the power level at 0.8 and the alpha level at 0.05. These parameters were established to detect large effect size differences between groups (ƞ^2^ > 0.5), based on a pilot study performed in our laboratory.

During the PET and MRI procedures, the animals were housed in pairs under a 12 h:12 h light-dark cycle, while maintaining the room temperature at 22 °C, and the humidity at approximately 50 %. During the EEG recording experiments, however, the animals were housed individually. Standard pellet food and water were provided *ad libitum*.

### Induction of the Temporal Lobe Epilepsy Model

According to the procedure of Hellier [31, 32], fifteen rats (epileptic group) received multiple systemic injections (IP) from 2.5 to 5 mg/kg of kainic acid (KA monohydrate ≥ 99 %, Sigma-Aldrich, USA) every 30 min, until they displayed several stage 5 generalized tonic-clonic seizures per hour (convulsive *SE*) [33]. Four rats were excluded from the experiment since they did not develop *SE*. The average KA dose per rat amounted to 18.5 ± 4 mg/kg. Simultaneously, a saline solution was administrated to five additional rats, which constitute the control group. *SE* was not interrupted with medication.

### Experimental Design

**Fig. 1:**
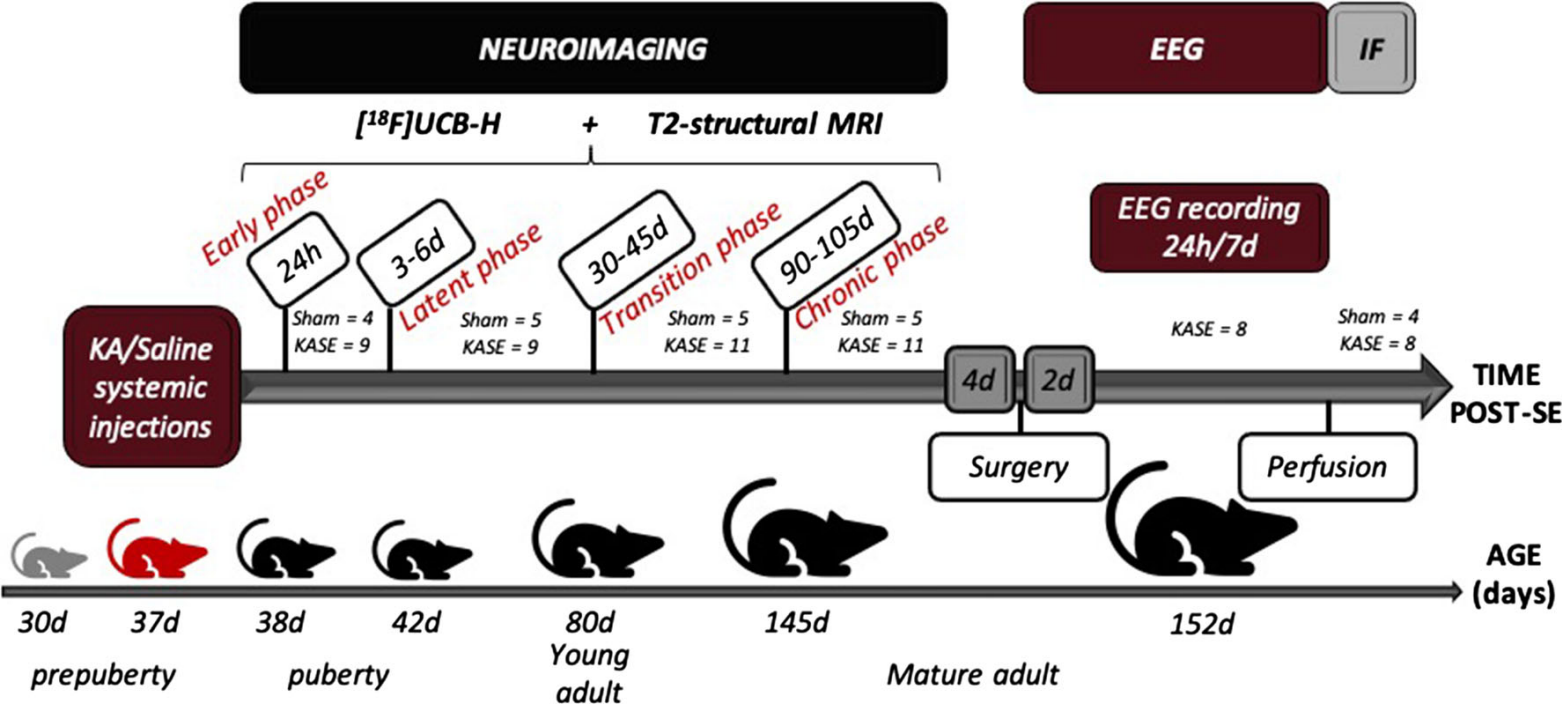
Experimental design. After the administration of multiple systemic doses of saline (control group) or kainic acid (KA, epileptic group), the animals were scanned at 4 time points, corresponding to the four phases of the TLE, with the [^18^F]UCB-H PET radiotracer, and with MRI. Subsequently, the EEG pattern of epileptic animals was recorded during 24 h per day, for 7 days. At the end of the last EEG measurement, all the rats were perfused, and the immunofluorescence labeling was performed to determine SV2A-expression levels. SE = status epilepticus; EEG = electroencephalography; IF = immunofluorescence.

Figure 1 presents the experimental design used in this study with all the different techniques, the number of animals employed, and the average age of the animals at the various measurement points.

Briefly, after the administration of KA (*n* = 11) or saline (*n* = 5), the animals were scanned at four time points, corresponding with the different phases of the TLE: (1) early phase (24 h), (2) latent phase (3 to 6 days), (3) transition phase (30 to 45 days), and (4) chronic phase (90 to 105 days). For all 4 phases, we performed [^18^F]UCB-H PET scans to quantify variations in SV2A, as well as structural T2-weighted MRI scans, to detect changes in brain structure. Four days after the last scan, a recording electrode was implanted into the hippocampus of the epileptic rats, and after 2 days of recovery, hippocampal EEG was continuously recorded for 1 week. On the last day of recording, rats of both groups were transcardially perfused and their brains were extracted. Then, immunofluorescence (IF) was performed to quantify the SV2A protein levels in the brain.

### PET and MRI Imaging

Before each scan session, the health of each rat was assessed. The animals that did not completely recover from the previous scans, or with a slight decrease in activity, unkempt hair coat or significantly thinner, were not scanned. Furthermore, the respiration rate and the rectal temperature were continuously measured during the PET and MRI scans, using a monitoring system. The temperature was maintained at 37 ± 0.5 °C using an air warming system (Minerve, France).

#### [^18^F]UCB-H Scans

The enantiomerically pure [^18^F]UCB-H was produced through one-step radiolabeling of a pyridyliodonium precursor in accordance with a reported method, obtaining a molar activity around 10 Ci/μmol [34].

The acquisition protocol was performed in different steps. First, the rats were anesthetized with a mixture of air with 30 % oxygen and isoflurane (4 % induction and 1.5–2 % for maintenance). Then, [^18^F]UCB-H (37 ± 4 MBq, 0.55 mL) was administered *via* the caudal vein (i.v.), and simultaneously, the PET emission data were recorded during 60 min in list mode on a Siemens FOCUS 120 microPET (Siemens, Knoxville, TN). Subsequently, a 10-min PET transmission scan was run in single event acquisition mode, using a ^57^Co source. The necessary information regarding data reconstruction and image specifications are detailed in previous works [35, 36].

#### MRI Scans

Following the PET acquisitions, the rats were transferred into the MRI (9.4T/310 ASR horizontal bore Agilent system), and anatomical T2-weighted brain images were obtained with a fast spin-echo multi-slice sequence, using the parameters detailed in [35, 36]. Owing to the small size of the rats during the two first phases, a surface rat coil was used in order to increase the signal-to-noise ratio (Rat Head Phased Array, 400 MHz, Rapid Biomedical GmbH, Würzburg, Germany).

#### Imaging Data Processing

The image data were processed using two software: SPM8 (Welcome Department of Cognitive Neurology, London, UK) and PMOD 3.6 (PMOD Technologies, Zurich, Switzerland).

#### [^18^F]UCB-H Quantification

A recently published methodology was used to quantify the uptake of the [^18^F]UCB-H radiotracer in the rat brain [35]. This methodology consists of using the standardized uptake value parameter (SUV), calculated on the average image corresponding to the 20 to 40-min time window after the radiotracer injection since this parameter is highly correlated with Vt in the case of the [^18^F]UCB-H radiotracer [35].

For each animal, the structural MRI image obtained at every phase was manually co-registered with the corresponding PET image, and subsequently, rigid body transformations were applied. Then, the obtained MRI image was spatially normalized into the PMOD structural MRI template. Finally, the inversed normalization parameters were calculated and applied to the *modSchiffer* atlas (Suppl. Fig. 1) in order to bring it into the individual PET space. From this atlas, created to adapt the PMOD’s rat atlas (W. Schiffer) to the morphology of the epileptic group, six ROIs were chosen: amygdala, striatum, hippocampus, thalamus, hypothalamus, and cortex. The radioactivity concentrations, extracted from the different ROIs, were expressed in SUV by means of its normalization by the injected dose and the animal body weight.

### EEG Surgery and Recordings

Surgery for EEG recordings was performed as described previously [37]: the epileptic rats were anesthetized with a mixture of air with 30 % oxygen and isoflurane (4 % induction and 2–2.5 % for maintenance). Additionally, 0.2 mg/kg of xylocaine was subcutaneously injected as local anesthesia. Small burr holes were drilled into the skull of the animals: eight for the positioning of anchor screws, one for an epidural screw electrode (above right frontal cortex), one for the reference/ground electrode (above left frontal cortex), and one for a custom-made polyimide-coated bipolar electrode (see Suppl. Fig. 2a). This bipolar recording electrode was placed in CA1 and in the *hilus* of the dentate gyrus using electrophysiological feedback (coordinates relative to Bregma: – 0.58 AP, + 0.40 ML, + 0.26/+ 0.35 DV, respectively). Immediately after surgery, the rats were injected subcutaneously with Metacam (2 mg/kg), and an ointment of Lidocaine and Neobacitracine was applied to the wound. Once finished the procedure, the rats were individually housed for 2 to 4 days before starting the EEG recording.

EEG data were analyzed with Matlab R2017a (The MathWorks, Inc., Natick, USA) by two experts, blinded to the PET results. The electrographic seizures were defined as described in [6, 37]: episodes of rhythmic spiking activity with a high amplitude (> 3 × baseline) and frequency (> 5 Hz), with a duration of, at least, 10 s (see Suppl. Fig. 2b). Three parameters were evaluated per animal: the number of electrographic seizures per week (*seizures/week*), their mean duration (*seconds/seizure*), and the total time in seizures (*total seconds*).

### Perfusion and Brain Pre-Treatments

At the end of 1 week of EEG recording, all the surviving rats of both groups were deeply anesthetized with pentobarbital (180 mg/kg, i.p.) and transcardially perfused with phosphate-buffered saline (PBS) followed by 4 % paraformaldehyde (PFA). The brains were extracted, post-fixed overnight at 4 °C in 4 % PFA solution, and cryoprotected for 3 to 5 days with increasing concentrations of sucrose (10 %, 20 %, and 30 %). Finally, the brains were frozen in isopentane at − 35 °C and serially sliced in 30 μm coronal sections with a cryostat (Leica, Germany). Slices from the striatum (Bregma, 1.92 to 1.20 mm) and the hippocampus (Bregma, − 2.64 to − 3.60 mm) were mounted onto coated glass slides (Premium Gelatin Subbed Slides, Southern Biotech MS, USA) and stored at − 80 °C.

### SV2A Immunofluorescence

Experiments were performed in duplicate, using a negative control to guarantee the primary antibody specificity for the SV2A protein. Additionally, glial fibrillary acidic protein (GFAP) immunofluorescence staining was used as a positive control for tissue quality.

The immunofluorescence staining procedure was performed as follows: firstly, slices were tempered, rinsed in PBS, and heated for 15 min in citrate buffer (pH 6.0) at 95 ± 3 °C for antigen retrieval. Thereafter, they were washed three times with a mixture of PBS and 0.05 % Tween20 (PBST, pH 7.5) and permeabilized for 10 min with 0.3 % Triton × 100. Slides were then incubated for 1 h with a blocking solution containing PBST and 10 % donkey serum (Sigma-Aldrich, St. Louis, USA). Immediately after, primary antibodies (polyclonal rabbit anti-SV2A, dilution 1:200; Abcam, Cambridge, MA; or polyclonal rabbit anti-GFAP, dilution 1:500, DAKO, Denmark) were applied overnight at 4 °C, in blocking solution. Following three washes with PBST, slides were incubated for 45 min with the secondary antibody (donkey anti-Rabbit Alexa Fluor 488-conjugated, dilution 1:500/1:1000 respectively; Thermo Fisher, OR, USA). Sections were washed with PBS, and nuclei were counterstained for 10 min with 300 nM DAPI in PBS (Thermo Fisher, OR, USA). Finally, slides were washed in PBS and distilled water and coverslipped with antifade medium (Fluoromount-G, Southern Biotechnology, Birmingham, AL, USA).

Confocal fluorescence images of the cortex (motor area), the caudate/putamen, and the different hippocampal structures (pyramidal cell layer of CA1 and CA3, and the *hilus* of the DG) were obtained using a scanning laser microscope (Leica TCS SP5 with AOBS, Leica Microsystems IR GmbH, Germany) with a × 20 objective. Images obtained with a × 10 objective were used only for presentation purposes. SV2A-immunoreactivity was quantified by measuring the optical density (OD) after background subtraction, using the ImageJ software (ver. 1.8.0, NIH).

### Statistical Analyses

Statistical analyses were carried out with SPSS (IBM® SPSS® Statistics 25; USA), setting the critical threshold of statistical significance at *p* < 0.05 for all the tests. Furthermore, the false discovery rate (FDR) was applied in all the statistical analyses to correct for multiple comparisons. Excel (2010) was used to graphically represent the results, expressed as the mean ± SEM.

#### [^18^F]UCB-H Data

The linear mixed-effects (MIXED) analysis and a restricted maximum likelihood (REML) estimation were used to longitudinally assess 1) changes in [^18^F]UCB-H uptake in control animals (*n* = 4–5) due to the brain development and 2) possible differences in [^18^F]UCB-H uptake between control and epileptic (*n* = 9–11) rats [38]. The assumptions of normality and homogeneity of residuals were previously evaluated with Levene’s and Kolmogorov-Smirnov tests. To challenge our hypothesis, significant effects and interactions between the variables *group* and *phases* were assessed *via* LSD test, followed by the FDR correction for multiple comparisons.

#### [^18^F]UCB-H and EEG Data

Since the three EEG parameters (*seizures/week*, *seconds/seizure*, and *total seconds*) were not normally distributed (Shapiro-Wilk test, *p* > 0.05), the Spearman’s rho method was employed to evaluate the relationship between these parameters and the [^18^F]UCB-H data for the chronic phase, in the epileptic group (*n* = 8).

#### SV2A Immunofluorescence

The independent samples *t* test (Student’s *t* test) was used to statistically compare SV2A-immunoreactivity between the two groups (control, *n* = 4; epileptic, *n* = 8). Additionally, the correlation between the OD and the [^18^F]UCB-H uptake (SUV) during the chronic phase was evaluated by means of Pearson’s *r* correlation coefficient.

## Results

### PET and MRI Imaging

#### [^18^F]UCB-H Results

**Fig. 2:**
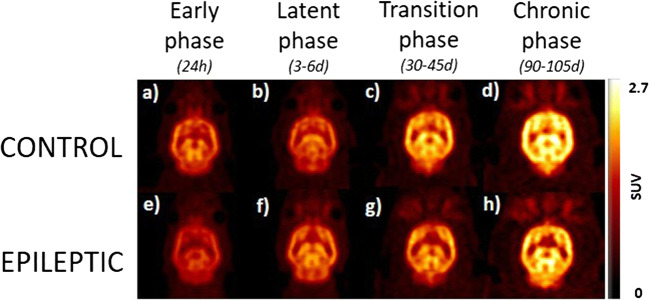
Representative images of longitudinal follow-up of variations in [^18^F]UCB-H uptake. The transaxial brain slices represent the [^18^F]UCB-H uptake of one representative animal of each group. Scans were performed at four time points, corresponding to the different phases of the TLE model.

The variations in [^18^F]UCB-H uptake (SUV) over time were analyzed in control (*n* = 4 to 5) and epileptic (*n* = 9 to 11) groups (see Fig. 2).

- Longitudinal variations of [^18^F]UCB-H uptake in control animals

The statistical analysis highlighted a significant increase in [^18^F]UCB-H uptake through the aging process, in all ROIs: hippocampus (F_3,4_ = 13.00; *p* < 0.05), amygdala (F_3,5_ = 14.98; *p* < 0.01), cortex (F_3,4_ = 10.80; *p* < 0.05), striatum (F_3,4_ = 11.62; *p* < 0.05), thalamus (F_3,5_ = 14.03; *p* < 0.01), and hypothalamus (F_3,8_ = 17.96; *p* < 0.01). Further pairwise comparisons showed that the differences between any of the first two (38 and 42 days old, puberty) and any of the last two (80 days old or young adults and 145 days old or mature adults) scans were statistically significant, with *p* < 0.05. In addition, in the amygdala and the hypothalamus, it was also possible to detect statistically significant differences between animals aged 80 (young adults) and 145 days (mature adults).

- Comparison of [^18^F]UCB-H uptake between control and epileptic animals

**Fig. 3:**
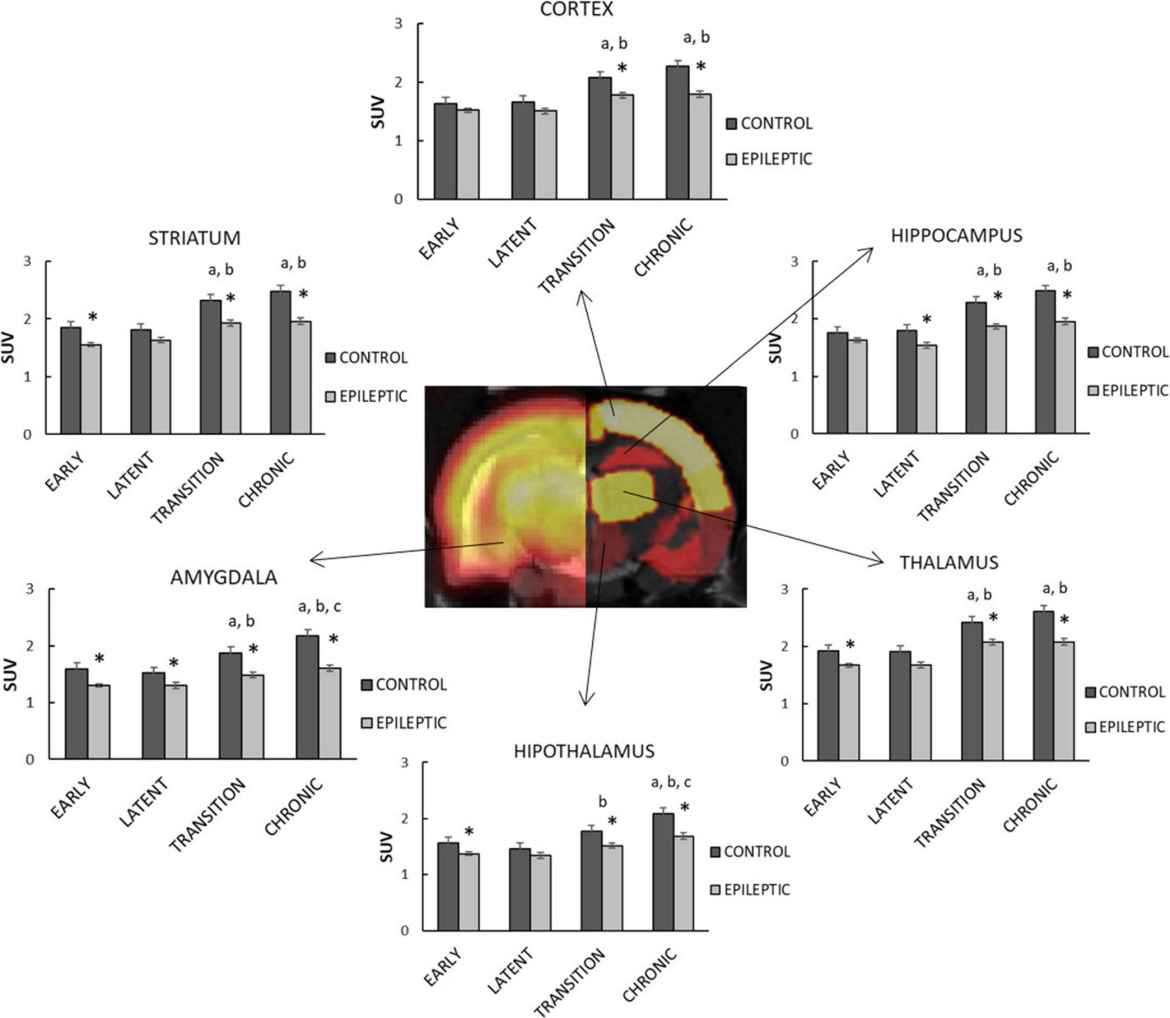
Quantification of variations in [^18^F]UCB-H uptake. The central figure represents the coronal view of a representative epileptic rat during the chronic phase (left hemisphere) and the Schiffer PMOD atlas (right hemisphere). Bar plots represent the [^18^F]UCB-H uptake (mean ± SEM) of control (*n* = 4–5) and epileptic groups (*n* = 9–11). Significant differences (*p* < 0.05) between one phase and the precedents are represented by letters, with: **a**= early phase (24 h), **b** = latent phase (3-6d), **c** = transition phase (30–45 days). Significant differences between groups are represented by stars.

The comparison between control and epileptic animals highlighted the existence of significant differences between both groups, showing the latter a decreased uptake in all the ROIs (Fig. 3): hippocampus (F_1,47_ = 47.46; *p* < 0.001), amygdala (F_1,37_ = 80.01; *p* < 0.001), cortex (F_1,46_ = 29.47; *p* < 0.001), striatum (F_1,43_ = 43.37; *p* < 0.001), thalamus (F_1,47_ = 40.72; *p* < 0.001), and hypothalamus (F_1,46_ = 30.71; *p* < 0.001). The mean difference between the control and the epileptic group increases from 12 ± 6 (early phase) to 22 ± 2 % (chronic phase), with the amygdala presenting the largest differences between groups. Further pairwise comparisons highlighted statistically significant differences between groups already during the early phase (24 h *post-SE*), in four regions: the amygdala (*p* = 0.002), the striatum (*p* = 0.002), the thalamus (*p* = 0.021), and the hypothalamus (*p* = 0.021). Interestingly, these group differences disappeared in the striatum, the thalamus, and the hypothalamus during the latent phase (3 to 6 days *post-SE*), while they appeared in the hippocampus (*p* = 0.023). At late phases (transition and chronic phase), the group differences in [^18^F]UCB-H uptake were global, with *p* < 0.001 in all the regions.

Finally, the interaction between the variables *group* and *phases* was not statistically significant in any of the regions (*p* < 0.05), which indicates that the epileptic animals presented an increase in [^18^F]UCB-H uptake through time equivalent to the one observed in control animals. However, this increase was lower in the epileptic group (22 ± 3 %) compared with the control one (around 36 ± 2 %).

#### MRI and Structural Brain Deformations

**Fig. 4:**
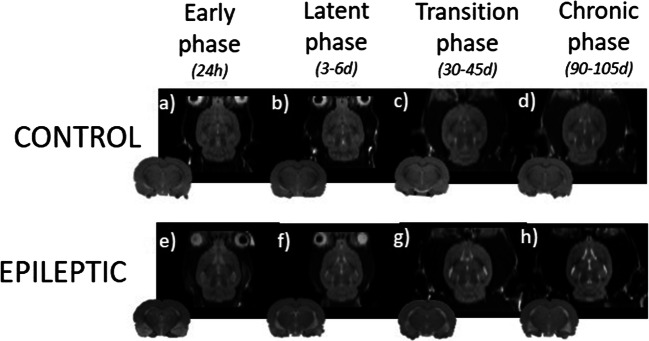
Longitudinal follow-up of alterations in the structure of the brain. Representative T2-structural MRI scanners, performed in two groups: **a**–**d** control rats, *n* = 4–5 and **e**–**h** epileptic rats, *n* = 9–11. The animals were scanned at four time points, corresponding to the different phases of the TLE model.

Structural brain alterations were present during the different phases of the epileptogenesis (Fig. 4): during the early phase, a T2* hyperintensity was detected in temporal regions like the amygdala and the entorhinal cortex, disappearing during the latent phase. During the transition and chronic phases, the progressive atrophy in the hippocampus and the amygdala is remarkable. Furthermore, we can observe a continuous increase in the ventricular CSF volume, which is more prominent in the lateral ventricles.

#### [^18^F]UCB-H and EEG

A strong inter-subject variability was seen in seizure parameters: the number of electrographic seizures per week ranged from 0 to 299, their mean duration ranged from 42 to 93 s, and the total time in seizures ranged from 0 to 12,139 s (see Table 1). However, the 2-tailed Spearman’s rho correlation did not reveal any significant correlation between these EEG parameters and the [^18^F]UCB-H uptake, with: 0.119 < *ρ* < 0.738, and *p* > 0.05.

**Table 1:** . Results of EEG evaluation in the epileptic group. The table presents the three parameters analyzed with EEG: the number of electrographic seizures per week (*seizures/week*), their mean duration (*seconds/seizure*), and the total time in seizures during the week of recording (*total seconds*), in the epileptic group (*n* = 8)

	Seizures/week	Seconds/seizure	Total seconds
Epileptic 1	0 seizures	0 s	0 s
Epileptic 2	1 seizure	99.6 s	99.6 s
Epileptic 3	2 seizures	42.4 s	84.7 s
Epileptic 4	4 seizures	93.3 s	373.3 s
Epileptic 5	79 seizures	47.9 s	4270.8 s
Epileptic 6	203 seizures	46.8 s	9982.2 s
Epileptic 7	238 seizures	51.4 s	11,441.5 s
Epileptic 8	299 seizures	47.9 s	12,138.7 s

#### SV2A Immunofluorescence

Two parameters were checked prior to performing the SV2A immunofluorescence staining: (1) the preservation of the brain tissue, evaluated by means of the astrocyte marker GFAP (Suppl. Fig. 3a), and (2) the specificity of the primary antibody against the SV2A protein (Suppl. Fig. 3b).

**Fig. 5:**
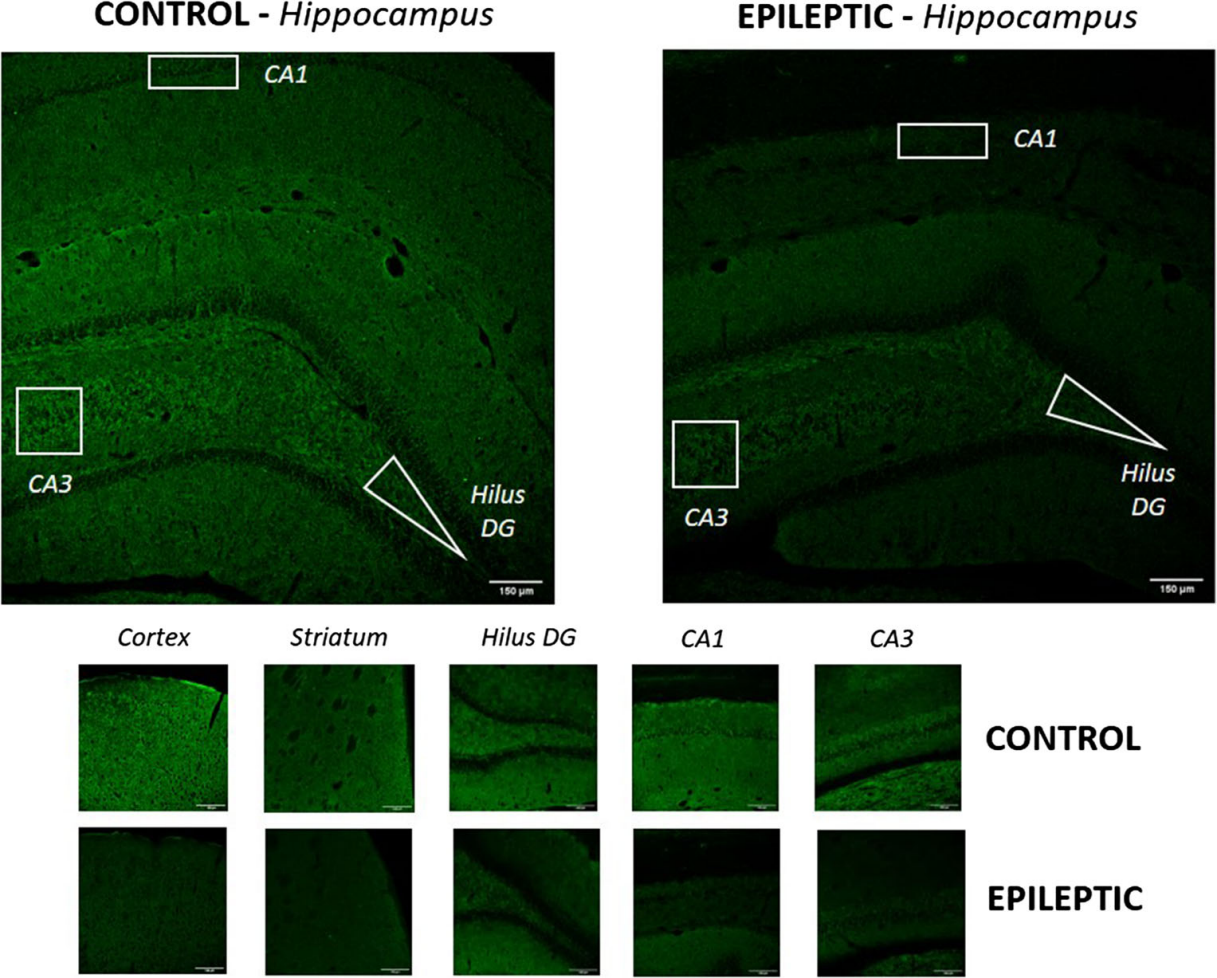
SV2A immunofluorescence. SV2A immunofluorescence staining was performed in control (*n* = 4) and epileptic rats (*n* = 9). We can observe a SV2A immunoreactivity decrease in the epileptic rat images, compared with the control ones. The scale bar (bottom right in all figures) represents 150 μm.

**Fig. 6:**
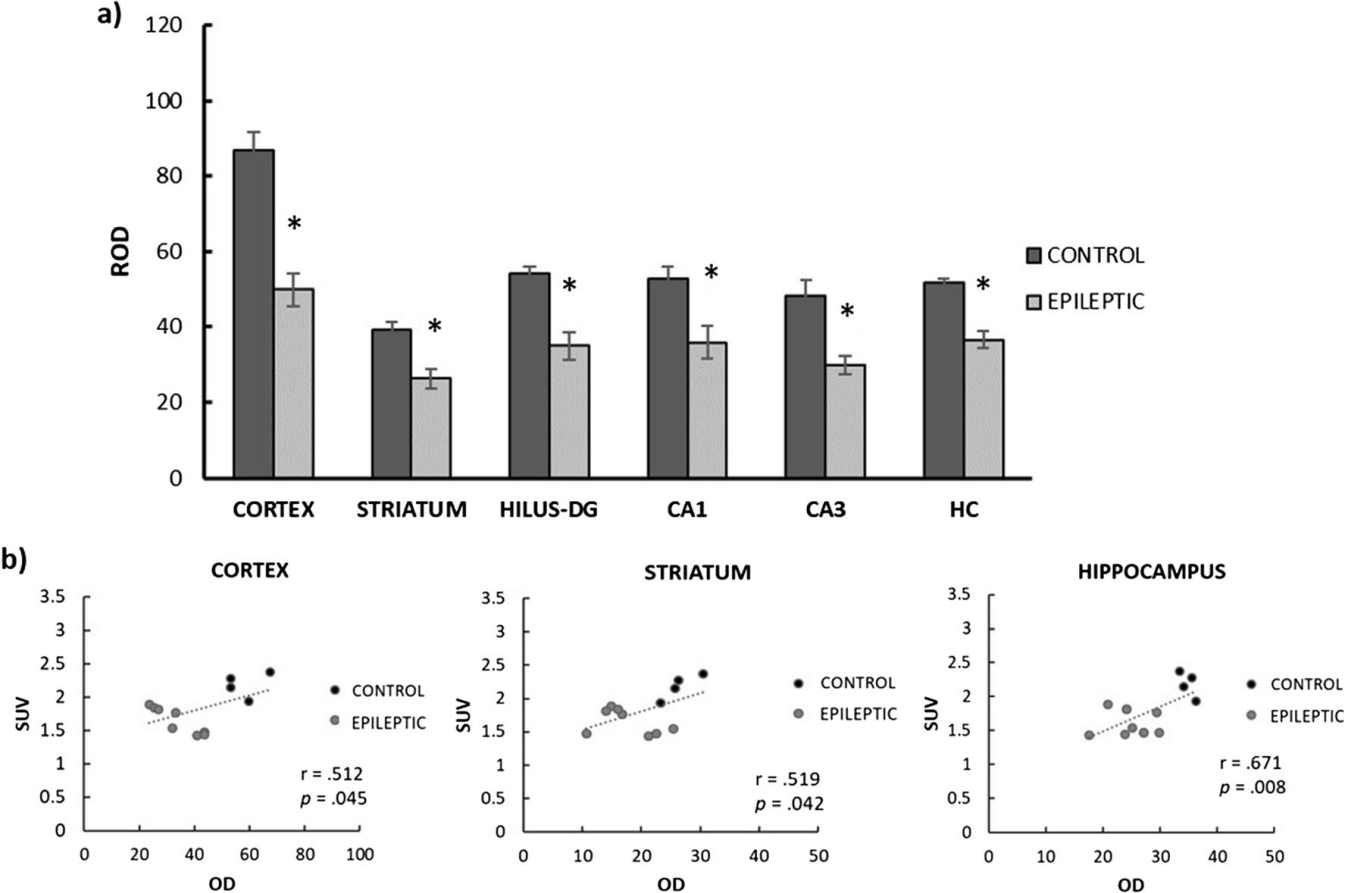
Immunofluorescence quantification of SV2A expression in different regions for control and epileptic groups. Bar plots (**a**) represent the mean ± SEM for control (*n* = 4) and epileptic groups (*n* = 9). Statistically significant differences in OD were found in all the regions (*p < 0.05). The scatter plots (**b**) illustrate regions with a significant correlation between the immunofluorescence (OD) and data obtained from [18F]UCB-H scanner (SUV). The dotted line represents the best fit for the data set. ROD = relative optical density; DG = dentate gyrus; CA = cornu ammonis; HC = hippocampus; OD = optical density.

Subsequently, we performed a SV2A immunofluorescence labeling (Fig. 5). The independent samples *t* test confirmed the existence of significant group differences (epileptic *vs* control) in all the regions analyzed (Fig. 6a): cortex (t (10) = 5.21; *p* < 0.003), striatum (t (10) = 3.22; *p* = 0.01), *hilus* of the *dentate gyrus* (t (10) = 3.48; *p* = 0.009), CA1 (t (10) = 2.59; *p* = 0.027), CA3 (t (10) = 4.08; *p* = 0.004), and in the average of all the hippocampal subregions (t (10) = 4.70; *p* = 0.003). The difference between groups ranged from 29.3 % in the hippocampus to 42.5 % in the cortex.

The relationship between the results obtained with the immunofluorescence (OD) and with the [^18^F]UCB-H radiotracer (SUV, chronic phase) was studied by means of the Pearson correlation. These results confirm a significant positive correlation between both parameters in all the ROIs (Fig. 6b): cortex (*r* = 0.512; *p* = 0.045), striatum (*r* = 0.519; *p* = 0.045), and hippocampus (*r* = 0.671; *p* = 0.024).

## Discussion

Understanding the pathological processes associated with epilepsy is key to the development of new biomarkers and treatments. In this study, we conducted, for the first time, a longitudinal *in vivo* evaluation of the variations of SV2A expression associated with the development of TLE in the kainic acid rat model. Additionally, to better understand the underlying neuropathological processes implicated in epileptogenesis, we assessed the relationship between the expression of this protein ([^18^F]UCB-H uptake) and the characteristics of the electrographic seizures. Before analyzing the results of this study, we confirmed the correct development of the TLE in the model used by means of two techniques: MRI and EEG. On the one hand, MRI images present structural brain deformations *post-SE*, similar to those reported in the existing literature [4, 39]. On the other hand, EEG showed clear electrographic hippocampal seizures. Both techniques, therefore, confirmed the correct development of the TLE model.

The results obtained with the [^18^F]UCB-H radiotracer highlight a significant increase in the SV2A rat brain expression from 38 (rat puberty) to 80–145 days old (rat adulthood). These results suggest an increase in this protein during the rat brain development which, to date, has only been evaluated from embryonic day E12 to postnatal day P30 [44]. Since SV2A is a presynaptic protein, this result could be reflecting an increase in the synaptic connections along the aging process.

Regarding the results about the comparison between the two groups, we can observe a lower increase in the SV2A expression in the epileptic group, compared with the control one. These group differences, some of them previously reported *in vitro* in both, animal models [40, 41] and TLE patients [40, 42, 43], can be detected *in vivo* in multiple regions as soon as 24 h *post-SE,* in all ROIs. Interestingly, in the hippocampus (epileptic *foci*) these differences are not present in the early phase, but they appear in the latent phase (3 to 6 days *post-SE*), increasing in the subsequent phases. This finding is consistent with one previous *in vitro* study [40]. Additionally, the longitudinal analysis reveals that the differences in SV2A expression increase progressively between the two groups, peaking at the chronic phase in the amygdala and the hippocampus (around 22 % difference). Furthermore, these group differences evolve differently depending on the evaluated ROI. For instance, the epileptic group presents a recovery in the SV2A levels during the latent phase (3 to 6 days *post-SE*) in four of the six regions: the amygdala, the striatum, the thalamus, and the hypothalamus. Such a recovery may be due to different compensatory processes taking place in response to *SE*, such as neurogenesis or synaptic reorganization [45, 46], and seems to be accompanied by the reabsorption of the oedema which appears in temporal regions during the acute phase, as we can observe in the MRI images.

In addition to the analyses of group differences in SV2A expression, we examined the possible relationship between variations in this protein and the presence of electrographic seizures. This evaluation did not reveal any significant correlation between the [^18^F]UCB-H uptake and the three EEG parameters measured: *seizures/week*, *seconds/seizure*, and *total seconds.* This result suggests that the variations in SV2A levels may impact the development of the TLE and the emergence of seizures, but not on the characteristics of the seizures (number and duration). However, since the number of animals employed is only suitable to detect large (and not medium or small) effect size differences, it would be necessary to increase the number of animals, in a future study, to perform a more precise evaluation about the relationship between the EEG measurements and the SV2A levels.

Finally, we employed the SV2A immunofluorescence to confirm these *in vivo* [^18^F]UCB-H results and validate our study. The statistically significant differences between groups (36 ± 7 %), along with the positive correlation between both SV2A measures, corroborate the suitability of [^18^F]UCB-H as an alternative to the *in vitro* techniques currently in use.

## Conclusions

In this paper, we have used the [^18^F]UCB-H radiotracer to detect variations in SV2A expression in the brain of a TLE rat model. Throughout this study, we have demonstrated its suitability to detect differences between control and epileptic animals and to follow-up on the brain maturation and the progression of the epileptic disease. [^18^F]UCB-H seems to be a promising tool to diagnose and monitor *in vivo* the epileptic disease in animal models and a valuable alternative to the *in vitro* immunofluorescence technique. Thus, the use of this radiotracer opens the door to new research which could help us to finally understand the role of the SV2A protein in epilepsy.

## Electronic supplementary material

ESM 1 (DOCX 258 kb)
