# Supplementary material for: Exploring with [18F]UCB-H the in vivo Variations in SV2A Expression through the Kainic Acid Rat Model of Temporal Lobe Epilepsy
Source: Mol Imaging Biol. 2020 Mar 23;22(5):1197–207. doi: 10.1007/s11307-020-01488-7 (PMC7497718; doi:10.1007/s11307-020-01488-7)
Supplement: Supplementary file 1 — (DOCX 258 kb) [file 11307_2020_1488_MOESM1_ESM.docx]

**SUPPLEMENTARY MATERIAL: Suppl Fig. 1-3**

**
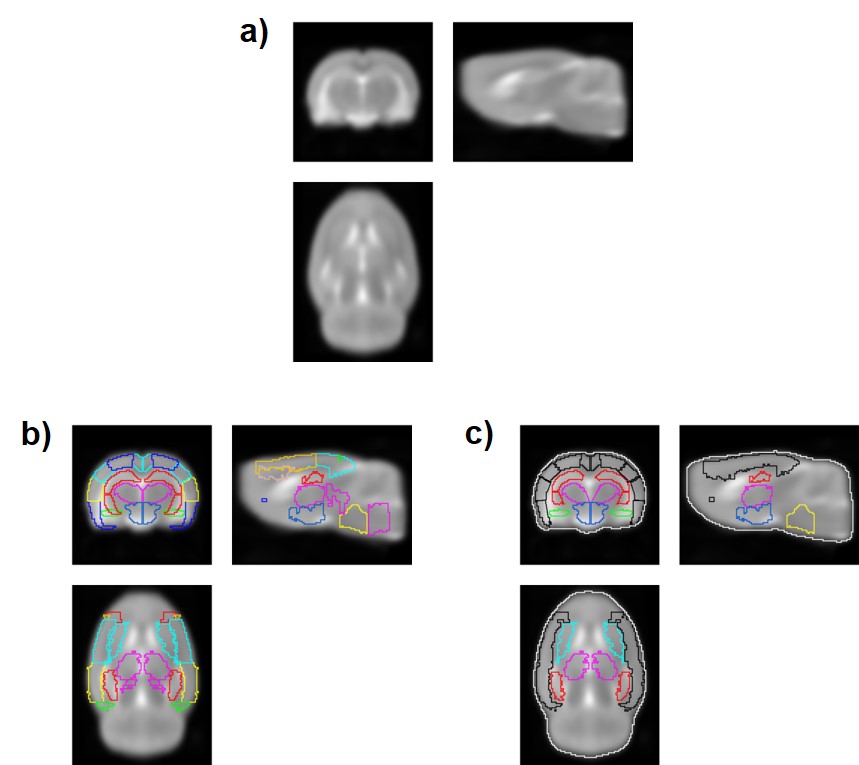
**

**Suppl. Fig. 1 Adjustment of Schiffer atlas.** The MRIs of the epileptic rats were normalized to bring them into the PMOD’s structural MRI image template (W. Schiffer-T2 rat template). Subsequently, we created an average image of the individual epileptic rats MRI (*avMRI* image) (**Suppl. Fig. 1a**). Then, the Schiffer atlas was superposed (**Suppl. Fig. 1b**) and manually adapted to the *avMRI* image by adjusting the different VOIs to the structural brain deformations of the *avMRI* image (e.g. ventricle enlargement) (**Suppl. Fig. 1c**). Finally, this new atlas was saved as *modSchiffer,* verifying its correct fitting to the individual MRI images of the epileptic group. The six selected ROIs can be observed in different colors: black = cortex; red = hippocampus; pink = thalamus; dark blue = hypothalamus; green = amygdala; light blue = striatum.

**
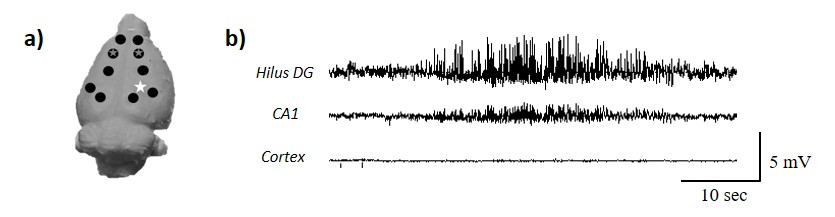
**

**Suppl. Fig. 2 Electrode and screws position, and example of a typical electrographic seizure.** **In Suppl. Fig. 2a**, black dots represent the position of the ten screws placed in the skull of the epileptic rats. The grey stars represent the screw electrode (right) and the reference/ground electrode (left). The white star represents the recording electrode, located in the right hippocampus. **Suppl. Fig. 2b** Represent a typical seizure event observed in an epileptic rat.


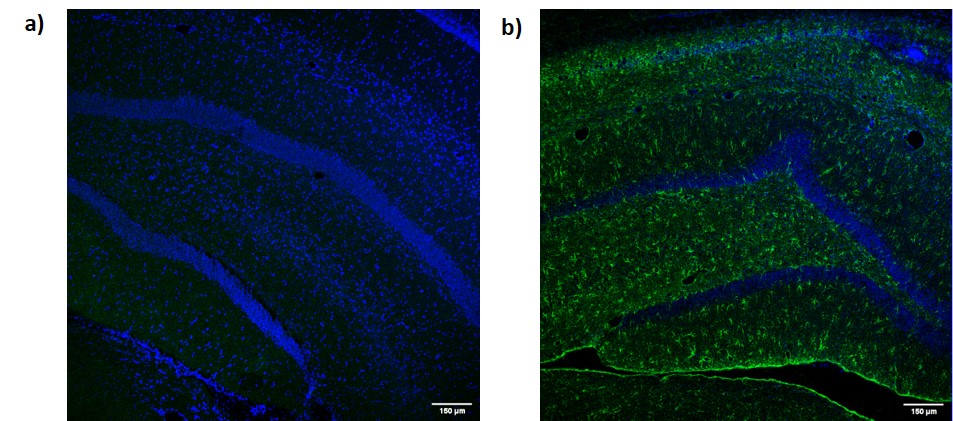


**Suppl. Fig. 3 Immunofluorescence controls.** Representative image of GFAP (green) and DAPI (blue) staining in an epileptic rat, used to verify the integrity of the brain tissue (**Suppl. Fig. 3a**), and SV2A non-specific uptake, obtained by incubating the slices only with the secondary antibody and DAPI (blue, **Suppl. Fig. 3b**).
